# Molecular approaches uncover cryptic diversity in intertidal *Ligia* isopods (Crustacea, Isopoda, Ligiidae) across the southern Africa coastline

**DOI:** 10.7717/peerj.4658

**Published:** 2018-04-19

**Authors:** Taylor M. Greenan, Charles L. Griffiths, Carlos A. Santamaria

**Affiliations:** 1 Biology Program, College of Science and Mathematics, University of South Florida Sarasota-Manatee, Sarasota, FL, United States of America; 2 Department of Biological Sciences and Marine Research Institute, University of Cape Town, Rondebosch, South Africa; 3 Department of Biological Sciences, Sam Houston State University, Huntsville, TX, United States of America

**Keywords:** South Africa biogeography, Oniscidea, Cryptic species, Ligiidae, Intertidal, Vicariance

## Abstract

Recent phylogeographic studies along the coastline of southern Africa have uncovered cryptic diversity in several coastal invertebrates, including direct developing crustaceans in the superorder Peracarida. These findings indicating the possible existence of additional cryptic diversity in other yet to be studied peracarids, particularly those known to harbor said cryptic diversity in other regions of the world. Isopods in the genus *Ligia* are one such taxon. They inhabit patchy rocky beaches, are direct developers, avoid the open water, and exhibit other biological traits that severely constrain their dispersal potential (e.g., poor desiccation resistance). These traits are thought to have led to long-term isolation of populations, and allopatric diversification in *Ligia* species around the world; however, *Ligia* species in southern Africa, where three endemic *Ligia* species of uncertain validity are known to exist, remain unstudied to date. In this study, we used mitochondrial and nuclear markers to characterize *Ligia* collected in 18 localities from Namibia to the KwaZulu-Natal region of South Africa. We report the presence of cryptic lineages within *Ligia* species in the region that suggest the need for taxonomic reevaluation of these isopod species.

## Introduction

Recent phylogeographic work on coastal invertebrate species has led to the discovery of cryptic diversity in poorly dispersing species around the world (e.g., Chan, Tsang & Chu, 2007; Hurtado, Lee & Mateos, 2013; Radulovici, Sainte-Marie & Dufresne, 2009; Santamaria et al., 2017; Santamaria et al., 2016; Santamaria, Mateos & Hurtado, 2014; Santamaria et al., 2013; Varela & Haye, 2012). In South Africa, cryptic diversity has been reported for several coastal invertebrate taxa (Evans et al., 2004; Mmonwa et al., 2015; Reynolds, Matthee & Von der Heyden, 2014; Ridgway et al., 2001; Teske et al., 2007; Zardi et al., 2007), including direct-developing crustacean peracarids. Teske et al. (2006) reported the presence of multiple deeply-divergent lineages for *Exosphaeroma hylecoetes* and *Iphinoe truncata*, with Baldanzi et al. (2016) reporting the presence of multiple evolutionary lineages within the amphipod *Talorchestia capensis*. These reports suggest other coastal peracarids in the region may harbor previously unreported cryptic diversity, underscoring the need for molecular characterizations of such organisms, so to better understand and delineate the biodiversity of coastal environments in southern Africa.

Coastal isopods of the genus *Ligia* have been shown to harbor cryptic diversity in other regions of the world. Although found along rocky coastlines throughout the world (Schmalfuss, 2003), the biology of these supralittoral isopods is marked by traits that severely limit their dispersal potential. As all other peracarids, they lack planktonic larvae with embryos developing inside a marsupium on females until hatching as fully-formed juveniles (termed manca). Adult *Ligia* isopods avoid open water and quickly attempt to regain the shore when submerged (Barnes, 1932; Barnes, 1935), exhibit low desiccation and submergence resistance (Barnes, 1936; Barnes, 1938; Todd, 1963; Tsai, Dai & Chen, 1997; Tsai, Dai & Chen, 1998; Zhang et al., 2016) and poor locomotion on non-rocky substrates. These traits limit both their overland and overwater dispersal potential, which may lead to severely restricted gene flow between populations, long term isolation, and in turn allopatric and potentially cryptic diversification, as reported for *L. hawaiensis* (Santamaria et al., 2013; Taiti et al., 2003), *L. exotica* and *L. cinerascens* (Hurtado et al., 2018; Yin et al., 2013), *L. occidentalis* (Hurtado, Mateos & Santamaria, 2010), *L. baudiniana* (Santamaria, Mateos & Hurtado, 2014), *L. oceanica* (Raupach et al., 2014), as well *L. vitiensis* and *L. dentipes* (Santamaria et al., 2017). Thus, molecular characterization of yet to be studied *Ligia* species may also uncover evidence suggestive of cryptic diversification.

One such case is that of *Ligia* populations along the southern Africa coastline. Currently, four valid *Ligia* species are thought to inhabit the region: the endemic *L. dilatata, L. glabrata*, and *L. natalensis*, and the introduced *L. exotica,* which to date is formally reported only from Durban harbour (Barnard, 1932). Of the endemic species, *L. dilatata* and *L. glabrata* were first described by Brandt (1833) from specimens collected in the ‘Cape of Good Hope’ (a vague term used by early researchers to describe any location in the then Cape Colony). The validity of these species was doubted by Collinge (1920), who suggested *L. glabrata* to be an immature form of *L. dilatata*. In the same work, Collinge described *L. natalensis* from specimens collected from Umhlali and Winklespruit Beach along the more subtropical coastline of KwaZulu-Natal. Later inspections by Jackson (1922) and Barnard (1932) determined all three species to be valid, based on differences in overall body shape, the shape of the stylet of the 2nd pleopod in males, and the length of the 2nd antenna. The first two of these traits have subsequently been shown to not be useful character for distinguishing amongst *Ligia* cryptic lineages (Santamaria et al., 2016; Santamaria, Mateos & Hurtado, 2014; Santamaria et al., 2013; Taiti et al., 2003), with similarities amongst *Ligia* species in southern Africa for these traits (see Fig. 2 of Barnard, 1932) making it unclear whether *Ligia* species in the region are valid taxa, or whether they harbor any cryptic diversity.

In this study, we aim to determine: (1) whether the currently accepted species of *Ligia* from South Africa represent reciprocally monophyletic clades, (2) whether these species harbor deeply divergent lineages that may represent cryptic species in need of taxonomic evaluation, and (3) the large scale distributional patterns of each of the *Ligia* species and lineages across southern Africa. To this end, we use characterized individuals collected from 18 localities spanning the area between Namibia and KwaZulu-Natal, using both mitochondrial and nuclear markers.

## Materials and Methods

### Field sampling, preservation, and identification

We hand-collected *Ligia* individuals from 18 localities around the coastline of southern Africa between 2014–2017. Detailed locality information is provided in Table 1. All samples were field-preserved and stored in 70% ethanol until molecular analyses were carried out. In the laboratory, specimens were identified to species by visual inspection of key characteristics (e.g., appendix masculina of the second pleopod of males) and comparing these traits to those reported for the various recognized *Ligia* species in southern Africa (Barnard, 1932; Ferrara & Taiti, 1979). Field collections were carried out under Scientific Collection Permit RES2017/53 issued by the South African Department of Environmental Affairs.

**Table 1:** Localities included and corresponding GenBank Accession Numbers for all genetic markers used, latitude, and longitude. Map labels correspond with other figures and tables.

Species	Locality	Map Label	*N*^a^	*N*_h_^b^	COI Acc. Nos.	NaK Acc. No.	Latitude	Longitude
*L. glabrata*	Luderitz, Namibia	A1	2	1	MH173093	MH173152	26°39′47″S	15°04′55″E
*L. glabrata*	Jacobsbaai, South Africa	A2	3	1	MH173096	N/A	32°58′26″S	17°53′06″E
*L. glabrata*	Ganzekraal, South Africa	A3	5	2	MH173094, MH173095	N/A	33°31′18″S	18°19′19″E
*L. dilatata*	Kommetjie, South Africa	B1	4	3	MH173097, MH173098, MH173099	N/A	34°08′17″S	18°19′24″E
*L. dilatata*	Koelbaai, South Africa	B2	4	2	MH173100, MH173101	MH173153	34°14′51″S	18°51′15″E
*L. dilatata*	Onrus, South Africa	B3	5	4	MH173103, MH173104, MH173105, MH173106	N/A	34°25′13″S	19°10′35″E
*L. dilatata*	Gansbaai, South Africa	B4	5	2	MH173102, MH173107	N/A	34°35′10″S	19°20′34″E
*L. dilatata*	L’Agulhas, South Africa	B5	10	4	MH173108, MH173109, MH173110, MH173111	N/A	34°49′26″S	20°01′01″E
*L. natalensis*	Knysna, South Africa	D1	5	4^c^	MH173126, MH173127, MH173128	N/A	34°02′16″S	23°01′09″E
*L. natalensis*	Skoenmakerskop, South Africa	C1	3	3	MH173143, MH173144, MH173145	N/A	34°02′45″S	25°38′01″E
*L. natalensis*	Summerstrand, Port Elizabeth, South Africa	C2	8	3	MH173142, MH173146, MH173147	MH173154	33°59′01″S	25°40′16″E
*L. natalensis*	Boesmansriviermond, South Africa	E1	4	2^c^	MH173112^c^, MH173119	N/A	33°40′51″S	26°39′20″E
*L. natalensis*	Kenton-on-Sea, South Africa	E2	10	7	MH173113, MH173114, MH173115, MH173116, MH173117, MH173118, MH173141	N/A	33°41′41″S	26°39′54″E
*L. natalensis*	Kidd’s Beach, South Africa	E3	10	5	MH173120, MH173122, MH173123, MH173124, MH173125	MH173155	33°08′50″S	27°42′10″E
*L. natalensis*	East London Harbor, South Africa	F1	5	4	MH173148, MH173149, MH173150, MH173151	N/A	33°01′28″S	27°53′26″E
*L. natalensis*	Salmon Bay, Port Edward, South Africa	D2	9	6	MH173121, MH173129, MH173130, MH173131, MH173132, MH173134	N/A	31°03′43″S	30°13′23″E
*L. natalensis*	Ivy Beach, Port Edward, South Africa	D3	9	1	MH173133	N/A	31°01′44″S	30°14′37″E
*L. natalensis*	Uvongo Beach, Margate, South Africa	D4	10	6	MH173135, MH173136, MH173137, MH173138, MH173139, MH173140	N/A	30°49′59″S	30°23′56″E

**Notes.**

a Number of individuals sampled in location.

b Number of unique COI haplotypes in location.

c Denotes haplotype shared by individuals in two populations.

### Molecular laboratory methods

We extracted total genomic DNA from several pleopods for 2–10 *Ligia* individuals per location using the Quick g-DNA MiniPrep Kit (Zymo Research, Irvine, CA, USA), following standard protocol instructions. For each individual, we PCR-amplified a 658-bp fragment of the Cytochrome Oxidase I (COI) mitochondrial gene using the LCO-1490 and HCO-2198 primers and previously published conditions (Folmer et al., 1994). We also PCR-amplified a 661-bp region of the sodium-potassium ATPase alpha subunit (NaK) gene using the NaKFb and NaKR2 primers and standard conditions (Tsang et al., 2008). Positive PCR amplifications were determined by visualizing PCR products on 1% agarose gels stained using SYBR Safe (Invitrogen, Carlsbad, CA, USA). Positive amplicons were sequenced at the University of Arizona Genetics Core, with sequences and assembled and edited (i.e., primer removal) using Geneious R8.0.5.

### Sequence alignments, phylogenetic analyses, and estimation of molecular divergence

The mitochondrial COI and nuclear NaK sequence datasets were aligned independently using the MAFFT server (Katoh & Standley, 2013) under standard settings for nucleotide sequences. Visual inspection of the resulting alignment produced no evidence suggestive of pseudogenes (e.g., stop codons, high rates of amino acid substitutions) or indels. Due to the limited phylogenetic signal within the NaK dataset, we did not concatenate the two datasets and carried out phylogenetic searches only on the COI resulting alignment. Relationships within the NaK dataset were estimated using haplotype network reconstructions.

We carried out preliminary phylogenetic analyses incorporating sequences produced in this study, as well as all publicly available COI sequences for other *Ligia* species and two *Ligidium* species (*Ligidium germanicum* and *Ligidium hypnorum*; COI accession numbers DQ182795 and DQ182781, respectively) so as to identify the most appropriate outgroup for our phylogenetic analyses. These preliminary analyses recovered the monophyly of southern Africa *Ligia* species; however, relationships amongst the ingroup were poorly resolved. As a result, we carried out our phylogenetic excluding outgroups and opting instead for rooting using a midpoint-root approach.

Prior to phylogenetic searchers, we determined the most appropriate model of nucleotide evolution for our COI dataset using the Modeltest script (Posada & Crandall, 2001) as implemented by FindModel (http://hiv.lanl.gov/content/sequence/findmodel/findmodel.html). Model selection was made by comparing the likelihood scores of all 28 models available based on an initial Weighbor tree. We then carried out phylogenetic searches under both Maximum Likelihood and Bayesian inference approaches. Maximum Likelihood phylogenetic searches were carried out in RAxML v8.1.2 (Stamatakis, 2014; Stamatakis, Hoover & Rougemont, 2008) and consisted of 1,000 thorough bootstrap replicates, followed by a thorough ML search under the GTR +Γ model. We produced a majority-rule consensus tree of all bootstrap replicates using the *Sumtrees* command of DendroPy v4.1.0 (Sukumaran & Holder, 2010).

We carried out Bayesian phylogenetic searches in MrBayes v3.2.5 (Ronquist & Huelsenbeck, 2003) and Phycas v2.2.0 (Lewis, Holder & Swofford, 2015). Searches in MrBayes consisted of two simultaneous searches of four chains, each sampled every 5,000th tree, while Phycas searches consisted of a single search, sampled every 50th tree. All Bayesian searches were carried out under the GTR +Γ model. For each Bayesian analysis, we estimated node support values by discarding all samples prior to stationarity (10–25% of sampled trees) and calculating a majority-rule consensus tree using the *Sumtrees* command of DendroPy v4.1.0 (Sukumaran & Holder, 2010).

Lastly, we used MEGA v7.0.7 (Kumar, Stecher & Tamura, 2016) to estimate COI Kimura 2-Parameter distances (K2P) within and amongst sampled localities and major lineages observed in the above phylogenetic reconstructions.

### Haplotype network reconstructions

We used the ancestral parsimony algorithm proposed by Templeton, Crandall & Sing (1992) as implemented in PopART v1.7 (Leigh & Bryant, 2015) to visualize relationships between all COI haplotypes recovered in this study. We estimated branch connections using the TCS network option (Clement, Posada & Crandall, 2000) of PopArt with networks considered separate if connections between them exceeded 33 steps (i.e., a 95% connection limit). We repeated this approach to visualize the relationships amongst NaK alleles.

### Molecular species delimitation analyses

We implemented molecular species delimitation analyses (hereafter MSDAs) using ABGD (Puillandre et al., 2012). This approach identifies putative species by producing pairwise differences amongst haplotypic data and identifying a putative inter-species gap in the distance distribution. Individual haplotypes are assigned to groups if their genetic distances are less than the proposed gap. Analyses were carried out on the COI data for each *Ligia* species separately using the ABGD server (http://wwwabi.snv.jussieu.fr/public/abgd/abgdweb.html) under standard settings, with the exception of the relative gap width (set to 1.0) and the distance setting (“Kimura (K80) TS/TV” = 2.0).

## Results

**Figure 1:**
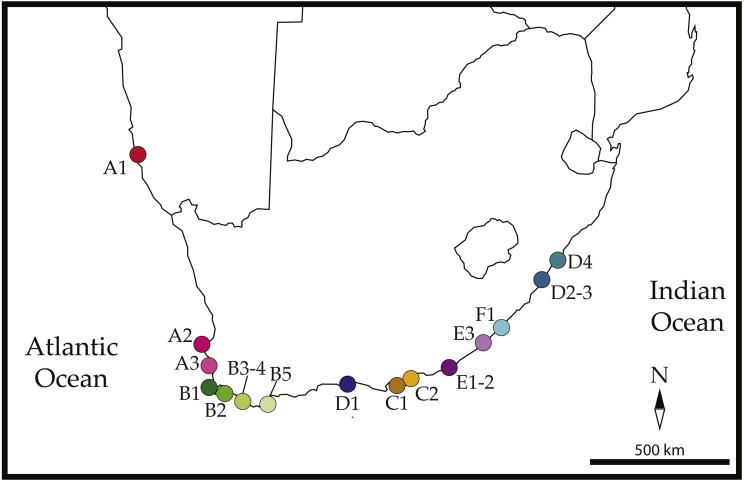
Sampled localities across southern Africa. Locations are as follows: (A1) Luderitz, (A2) Jacobsbaai, (A3) Ganzekraal, (B1) Kommetjie, (B2) Koeelbai, (B3) Onrus, (B4) Gansbaai, (B5) L’Agulhas, (C1) Skoenmakerskop, (C2) Summerstrand, (D1) Knysna, (E1, E2) Boesmansriviermond and Kenton-on-Sea, (E3) Kidd’s Beach, (F1) East London Harbor, (D2–D3) Salmon Bay and Ivy Beach, (D4) Uvongo Beach. Colors and labels correspond to those used in all other figures and tables. Map is edited from a Wikimedia figure by Hosie published under a CC license: https://commons.wikimedia.org/wiki/File:SubSaharanAfrica.svg.

We successfully amplified 658-bp of the COI mtDNA gene for 111 *Ligia* individuals from 18 localities across southern Africa (Fig. 1): 10 that were identified as *L. glabrata*, 28 as *L. dilatata*, and 73 as *L. natalensis*. We recovered a total of 59 COI haplotypes, which were separated by 162 parsimony informative sites. All new COI haplotypes and NaK alleles recovered in this study have been deposited in GenBank under accession numbers MH173093–MH173155 (Table 1).

### Phylogenetic results

**Figure 2:**
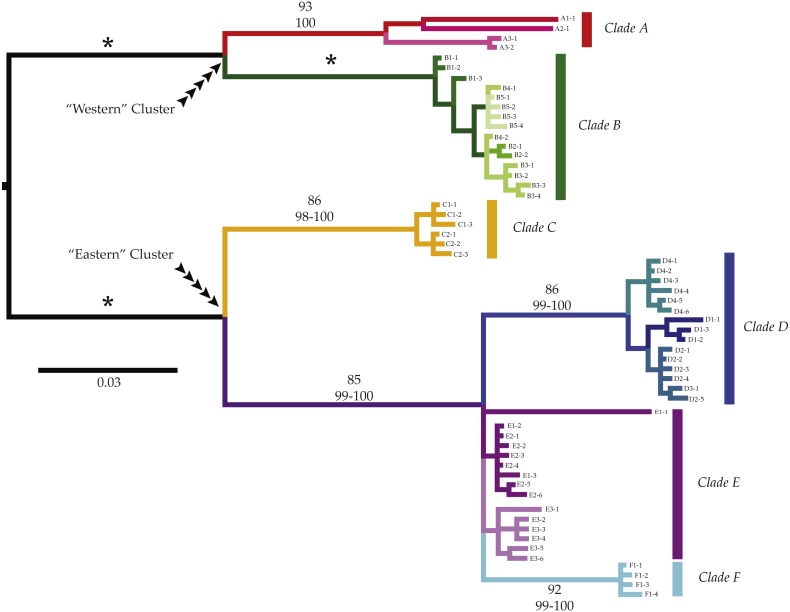
Phylogenetic patterns of *Ligia* from southern Africa. We observed three monophyletic groups that largely match currently valid species of *Ligia* in southern Africa; however, additional genetic divergence was observed within some of these groups. Six major clades were observed (*Clade A*: reds; *Clade B*: greens; *Clade C: yellows*; *Clade D*: blues; *Clade E*: purples; *Clade F:* cyan) containing seven moderately to highly divergent lineages. Most of the lineages contained haplotypes from geographically nearby localities. Clades and lineages exhibit mostly disjunct geographic distributions matching biogeographic regions; however, exceptions exist. Values above branches represent support values for the corresponding branch (top value: Bootstrap Support; bottom: Maximum Posterior Probablities; *: 100 in all analyses).

We observed a basal split between two well supported clusters of highly divergent clades in all analyses: a “Western” cluster (reds and greens in all figures; Bootstrap support (BS): 100; Maximum Posterior Probability (MPP): 100%; Fig. 2) with a geographic distribution from Namibia to the Cape Agulhas region, and an “Eastern” cluster (blues, yellows, and purples in all figures; Bootstrap support (BS): 100; Maximum Posterior Probability (MPP): 100%) that was distributed from Knysna, on the south coast of South Africa (hereafter SA), to the KwaZulu-Natal region of SA. Each of these clusters was composed of two or more highly divergent clades (clades *A*–*F*; amongst clade COI K2P divergences 3.1–17.2%, Table 2).

COI haplotypes assigned to the “Western” cluster were further divided into two highly divergent clades (amongst clade COI K2P divergences: 8.5–10.7%, Table 2). *Clade A* (reds in all figures; BS: 93%; MPP: 100%) included all *Ligia* individuals sampled in Namibia (A1), as well as from two locations in SA: Jacob’s Bay (A2) and Ganzekraal (A3) and corresponds to the species morphologically identified as *L. glabrata*. Within this clade, we observed three lineages that correspond with the sampled localities and that were moderately divergent from each other (COI K2P: 5.1–5.6%; Table 3). The relationships between these lineages were not well supported; however, our analyses suggest a sister-taxon relationship between the lineage found in *Ligia* from Luderitz, Namibia (A1) and that found in Jacob’s Bay (A2) (BS, MPP < 60). The second clade part of the “Western” cluster, *Clade B* (greens in all figures; BS: 100; MPP: 100), comprised all *Ligia* individuals collected from localities between the Cape of Good Hope and Cape Agulhas (B1–B5) and morphologically corresponds to the species *L. dilatata*. *Clade B*, contrary to the *Clade A*, does not appear to be composed of any further divergent lineages and within-clade divergences within it were low (COI K2P: 0.0–1.2%; Table 2).

**Table 2:** Pairwise amongst clade COI K2P divergences. Ranges represent minimum and maximum values obtained when comparing individuals amongst clades, with values in parenthesis representing average divergences between members of various clades.

	*Clade A*	*Clade B*	*Clade C*	*Clade D*	*Clade E*	*Clade F*
*Clade A*	0.0–5.6% (3.7%)					
*Clade B*	8.5–10.7% (9.4%)	0.0–1.2% (0.5%)				
*Clade C*	13.2–15.3% (14.1%)	13.3–14.6% (13.8%)	0.0–1.1% (0.4%)			
*Clade D*	14.9–16.8% (15.7%)	15.4–17.0% (16.2%)	10.3–12.0% (11.2%)	0.0–1.9% (0.7%)		
*Clade E*	14.3–17.2% (15.4%)	15.1–16.6% (15.6%)	9.4–12.1% (10.0%)	3.5–6.3% (4.5%)	0.0%–5.4% (1.3%)	
*Clade F*	15.1–16.9% (15.6%)	15.5–16.5% (15.9%)	11.0–12.2% (11.5%)	3.6–6.4% (4.1%)	3.1%–6.4% (3.8%)	0.0–0.6% (0.4%)

**Table 3:** Pairwise divergences for localities/lineages from Clade A as determined by COI K2P. Ranges represent minimum and maximum values obtained when comparing individuals from different sampling localities, with values in parenthesis representing average divergences between members of said localities.

	A1	A2	A3
A1	0.0–0. 0% (0.0%)		
A2	5.6–5.6% (5.6%)	0.0–0. 0% (0.0%)	
A3	5.1–5.2% (5.2%)	5.2%–5.4% (5.2%)	0.0–0.2% (0.1%)

**Figure 3:**
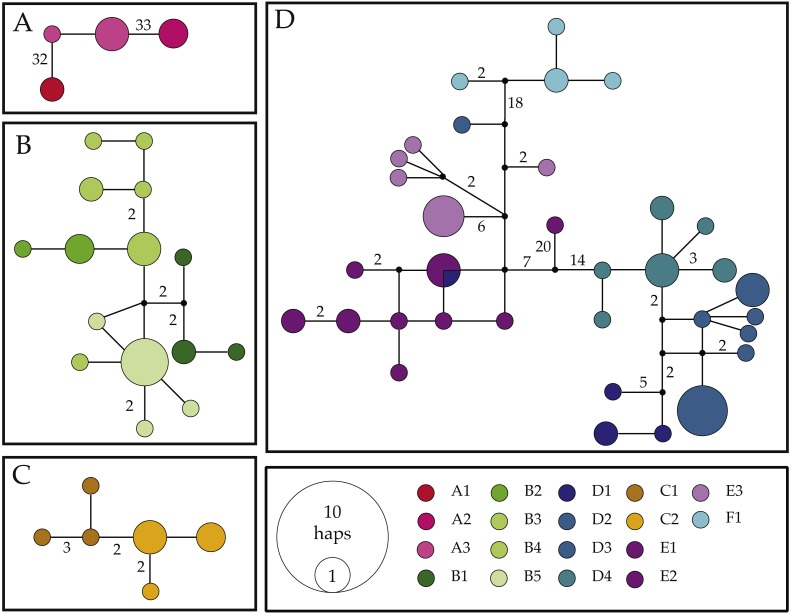
Haplotype networks for the COI mitochondrial gene fragment of *Ligia* from southern Africa. Colors correspond with those used in other figures. Black circles represent inferred unsampled haplotypes with numbers along branches showing number of nucleotides differences between haplotypes. Frequency of haplotype recovery is represented through the relative sizes of the circles. (A–D) represent networks which are more than 5% different. Locality labels correspond with those in Fig. 1 and Table 1.

The “Eastern” cluster, which contained all *Ligia* collected from Knysna to the KwaZulu-Natal region of South Africa, was composed of three highly divergent and well supported monophyletic clades (*C–E*; COI K2P 3.1–12.2%; Table 2) which morphologically correspond to the established species *L. natalensis*. Within this cluster, clades *D*, *E,* and *F* (blues and purples in all figures) are placed in a well-supported clade (BS: 82; MPP: 100) with *Clade C* (yellows in all figures) in turn sister to this group. Relationships between *D*, *E*, and *F* are not well resolved. *Clade C* containing all *Ligia* individuals collected in the Port Elizabeth area (C1–2), was highly supported across analyses (BS: 86; MPP: 98–100), and exhibited low within-clade divergences (COI K2P 0.0–1.1%, Table 2). This clade was highly divergent from all other clades in the “Eastern” cluster (COI K2P 9.4–12.2%; Table 2) and appears genetically distinct enough to be considered a separate and previously unrecognized species within the *natalensis* group. *Clade D* (BS: 86; MPP: 99–100) includes most COI haplotypes obtained from *Ligia* individuals collected in Knysna (D1) and the Port Edward area (D2–4). Within clade divergence for *Clade D* was low (COI K2P 0.0–1.9%, Table 2). *Clade E* contained all COI haplotypes recovered from individuals from the Kenton-on-Sea area (E1–2), those from Kidd’s Beach (E3), as well as one each from Knysna (D1) and Salmon Bay (D2). Although this clade was not strongly supported by any phylogenetic analyses (BS, MPP < 50), we denote it as a separate clade given the very low levels of divergence between all haplotypes in it (COI K2P average ∼1.3%; Table 2), moderate amongst clade divergence when compared to haplotypes from clades *D* and *F* (COI K2P 3.5%–6.4%; Table 2), and the results of haplotype network reconstructions (Fig. 3). Lastly, the well supported *Clade F* (cyan in all figures, BS: 92; MPP: 99–100) contained all individuals collected at the East London Harbor (F1) and exhibited low levels of within clade divergence (COI K2P 0.0–0.6%; Table 2).

### COI haplotype network reconstructions

The results of our COI haplotype network reconstructions (Fig. 3) largely match patterns produced by our phylogenetic analyses, as we recovered four separate networks (i.e., connections of <95%) largely corresponding to clades observed in phylogenetic reconstructions.

*Network I* (Fig. 3A) contained four haplotypes recovered from *Ligia* individuals from Luderitz (A1), Jacob’s Bay (A2) and Ganzekraal (A3). In Luderitz, we recovered a single haplotype that diverged by 32–33 steps from haplotypes recovered from Ganzekraal, which in turn diverged by 33–34 steps from the single haplotype recovered in Jacob’s Bay. This network closely parallels the patterns observed for *Clade A* in our phylogenetic reconstructions and contains all individuals morphologically identified as *L. glabrata*.

*Network II* (Fig. 3B) contained all 15 haplotypes recovered from *Ligia* collected between the Cape of Good Hope and Cape Agulhas (B1–B5), closely matches *Clade B*, and include all individuals identified as *L. dilatata*. Divergences in this network were low, with most connections between haplotypes being only 1–2 steps and the maximum connection between haplotypes being 10 steps. Despite such short connections, the network suggests some isolation between localities, as no sharing of haplotypes is apparent. Furthermore, haplotypes recovered within a single location appear to be much more similar (1–2 steps) than to those found at other locations in the region (∼4 steps).

*Network III* (Fig. 3C) consisted of six haplotypes recovered from *Ligia* collected in localities near Port Elizabeth (C1–2) and corresponds with *Clade C* from our phylogenetic findings. As observed in *Network II*, connections between haplotypes are very short, as most haplotypes are connected by <3 steps and the maximum span between haplotypes is seven steps. Morphologically, all members of this network were identified as *L.  natalensis*.

Lastly, *Network IV* (Fig. 3D) contained 34 haplotypes divided into three sub-networks separated by <18 steps. These sub-networks appear to correspond with clades *D–F* from our phylogenetic results and include all other *L. natalensis* individuals. One sub-network (blues in Fig. 3) contained all but two haplotypes from localities around Knysna (D1) and Port Edward (D2–4). Another sub-network (purples in Fig. 3) contained all the haplotypes collected in the localities of Kenton-on-Sea (E1–2) and Kidd’s Beach (E3). Intermediate to these two subnetworks is a small subnetwork of four haplotypes recovered from individuals collected in East London (F1; cyan in Fig. 3). In general, haplotypes collected from the same locality are much more similar to each other (<6 steps) than those from other localities (>10 steps), with two exceptions. A COI haplotype recovered from a *Ligia* individual collected in Salmon Bay (D2) was much more similar to those found in Kidd’s Beach (E3; 4–9 steps) than others from its own location (>29 steps). This haplotype was not observed in any other *Ligia* individual from any other locality. The other exception was a COI haplotype collected from an individual collected in Knysna (D1) that was shared with individuals from the Kenton-on-Sea area (E1–2). These patterns are congruent with the amongst-locality divergences where these lineages were found (Tables 4 and 5).

**Table 4:** Within Clade divergences for populations from *Clade D* as determined by COI K2P. Ranges represent minimum and maximum values obtained when comparing individuals from different sampling localities, with values in parenthesis representing average divergences between members of said localities.

	D1	D2	D3	D4
D1	0.0–4.6% (2.0%)			
D2	0.8–4.8% (1.9%)	0.0–4.8% (1.2%)		
D3	0.8–4.1% (1.6%)	0.3–4.6% (0.9%)	0.0–0.0% (0.0%)	
D4	0.9–4.1% (1.8%)	0.5–4.3% (1.2%)	0.8–1.2% (1.0%)	0.0–0.8% (0.4%)

**Table 5:** Within Clade divergences for populations from *Clade E* as determined by COI K2P. Ranges represent minimum and maximum values obtained when comparing individuals from different sampling localities, with values in parenthesis representing average divergences between members of said localities.

	E1	E2	E3
E1	0.0–0.5% (0.3%)		
E2	0.2–4.7% (0.9%)	0.0–4.9% (1.2%)	
E3	0.8–1.7% (1.2%)	0.8–5.4% (1.8%)	0.0–1.4% (0.8%)

### NaK haplotype network reconstructions

**Figure 4:**
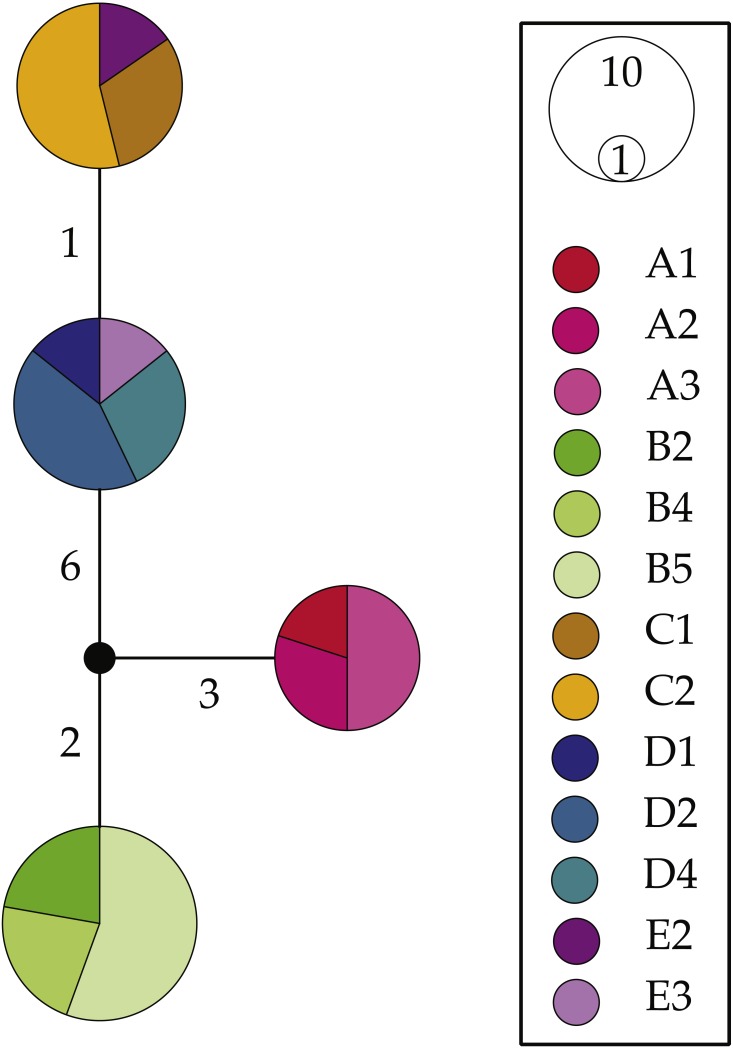
Haplotype networks for the nuclear gene NaK for *Ligia* from southern Africa. Colors correspond with those in all other figures with locality labels corresponding with those in other figures and Table 1. Unsampled or missing alleles are denoted by empty circles with numbers along branches indicating number of mutational steps separating alleles. Circle sizes and color proportions within them are relative to allele frequencies.

NaK haplotype network reconstructions (Fig. 4) were congruent with the above results; however, they produced much simpler patterns. We uncovered four NaK alleles separated by 1–10 steps: one allele shared by all surveyed individuals within *Clade A* (*L. glabrata*), one shared by all individuals within *Clade B* (*L. dilatata*), and two alleles from individuals from within the “Eastern” Cluster (*L. natalensis*). These latter two alleles were much more similar to each other (1 step) than to the other two recovered alleles (7–9 steps). The alleles found in the other clades were also highly divergent from those found in other clades, with the *Clade A* allele separated by 5–10 steps from other alleles and the one found in *Clade B* being separated by 5–9 steps. These patterns are largely concordant with those produced by mitochondrial analyses when considering the low levels of variation of this marker.

### Molecular species delimitation analyses

MSDAs suggested the presence of cryptic species for all three nominal *Ligia* species in the region. ABGD identified three putative species for *L. glabrata* individuals regardless of the prior maximal distance (i.e., barcode gap) assumed, with groups corresponding with the locations in which these individuals were found (Group 1: A1, Group 2: A2, Group 3: A3). For *L. dilatata* two groups were identified (Group 1: B1, Group 2: B2–5); however, this split was based on a prior maximal distance of only 0.001668. Another two putative species were identified for *L. natalensis* individuals, with those from C1 and C2 placed in Group 1, and those from D1–4, E1–3, and F1 placed in Group 2. These results were based on prior maximal distances of 0.02154 and above. Exclusion of the Port Elizabeth individuals (C1–2) from ABGD analyses did not produce any additional groupings within the *L. natalensis* individuals.

## Discussion

Three currently valid *Ligia* species are thought to be endemic to the southern Africa coastline (Schmalfuss, 2003); however, results reported herein suggest this may underrepresent the biodiversity of these isopods in the region. Although phylogenetic reconstructions place individuals putatively identified to nominal southern Africa species in well-supported and highly divergent reciprocally monophyletic clades, the clades composed of individuals putatively identified as *L. glabrata* (*Clade A*) and *L. natalensis* (the “Eastern” cluster) are each comprised of several moderately to highly divergent lineages. For instance, within the “Eastern” cluster we observe a deep split between *Clade C* and all other lineages in the cluster, with divergences amongst these lineages matching and/or exceeding those seen for pairwise comparisons between *L. glabrata* and *L. dilatata* (8.5–10.7% COI K2P, Table 2), as well as those previously reported for other *Ligia* species pairs, such as *L. perkinsi/L. hawaiensis* (11.9–16.7% COI K2P; Santamaria et al., 2013). We also report moderate levels of divergence amongst the other lineages within the “Eastern” cluster (i.e., *D*, *E,* and *F;* COI K2P 3.1–6.4%; Table 2), as well as amongst the three highly divergent and geographically disjunct lineages recovered within *Clade A* (COI K2P 5.1–5.6%; Table 3). The divergences observed amongst these lineages exceed intra-species levels of divergence reported for invertebrate species (Hebert et al., 2003) and match those reported for other potential cryptic species in other *Ligia* isopods (Hurtado, Lee & Mateos, 2013; Hurtado et al., 2014; Hurtado, Mateos & Santamaria, 2010; Santamaria, Mateos & Hurtado, 2014; Santamaria et al., 2013; Xavier et al., 2012). Relatedly, ABGD analyses suggest the presence of 2–3 cryptic species in each of the three nominal *Ligia* species in the region, as suggested by the presence of a barcode gap amongst individuals from different locations.

Past research on *Ligia* from other regions suggest that the currently valid *Ligia* species in southern Africa may be cryptic species complexes in need of taxonomic revision. Hurtado, Mateos & Santamaria (2010) reported the presence of seven major clades (amongst clade divergences: 7.3–29.9% COI K2P) in the area from Central California to Central Mexico, an area thought to harbor the single endemic species *L. occidentalis*. Reciprocal crosses between localities now known to harbor highly divergent populations done by McGill (1978); however, failed to produce viable offspring, suggesting that some of the lineages reported by Hurtado, Mateos & Santamaria (2010) represent true biological species. A possible cryptic species complex has also been reported from the Hawaiian archipelago, where Santamaria et al. (2013) found evidence suggesting the lone intertidal *Ligia* species endemic to the islands is a paraphyletic complex of at least four highly-divergent lineages (amongst clade divergences: 10.5–16.7% COI K2P). These previous findings, combined with those reported herein, thus suggest the need for taxonomic evaluations of *Ligia* isopods from southern Africa with our MSDAs results serving as taxonomic hypotheses (e.g., Boissin et al., 2017).

Any future taxonomic work would be enhanced by the inclusion of additional populations along the southern Africa coastline. Although our sampling spans ∼2,400-km of coastline from Namibia to South Africa, three large coverage gaps exist in our sampling: ∼750-km along the west coast between Jacobsbaai and Luderitz (Namibia), ∼370-km along the south coast between Cape Agulhas and Knysna, and the section of the east coast from Durban northwards to the Mozambique border. Sampling these areas may help determine whether yet to be sampled *Ligia* populations harbor additional cryptic lineages and elucidate the geographic distribution of *Ligia* species and lineages in the region. The latter is of importance, as the distributional patterns reported herein show some slight departures from the reported distributions for *Ligia* species in southern Africa. According to Ferrara & Taiti (1979) and Schmalfuss (2003), *L. glabrata* and *L. dilatata* both occur from Namibia (Luderitzbucht) to the Cape Peninsula (CP), with the former extending its range to west shores of the CP and Dyer Island and the latter extending to the east shores of the CP and Dassen Island. Our results; however, suggest that the distribution of *L. glabrata sensu lato* extends from Namibia to north of Cape Town, with *L. dilatata s. l.* occurring from the area surrounding Cape Town to Cape Agulhas. Meanwhile, *L.  natalensis* is reported to occur from Victoria Bay to KwaZulu-Natal (Ferrara & Taiti, 1979; Schmalfuss, 2003), which is congruent with our findings.

As we lack the fine-scale sample coverage needed to confidently determine the geographic extent of species and lineages herein reported, the distributional patterns described above should be seen as preliminary broad-scale descriptions in need of further validation. As our sampling efforts consisted of a single visit to each site, future sampling should include not only new localities but also the sampling of different microhabitats or tidal levels at localities included herein. This will not only help determine the geographic ranges of *Ligia* lineages and species in the region, but also help determine whether lineages and species are sympatric or truly allopatric.

## Conclusions

By using morphological identifications as well as nuclear and mitochondrial markers to characterize 18 *Ligia* populations from southern Africa, we report patterns that suggest the biodiversity of these isopods is under-reported in this region. Our findings are in line with reports of allopatric genetic differentiation across *Ligia* species from other regions (Eberl et al., 2013; Hurtado, Mateos & Santamaria, 2010; Hurtado et al., 2018; Raupach et al., 2014; Santamaria et al., 2017; Santamaria, Mateos & Hurtado, 2014; Santamaria et al., 2013; Taiti et al., 2003; Yin et al., 2013), as well as reports of cryptic diversity within other coastal invertebrates along the coastline of South Africa (Baldanzi et al., 2016; Evans et al., 2004; Ridgway et al., 2001; Teske et al., 2006; Teske et al., 2007; Zardi et al., 2007). The presence of several cryptic lineages within nominal *Ligia* species in southern Africa suggest the possible presence of putative cryptic species in the area, underscoring the need of taxonomic evaluation to determine whether these lineages are indeed valid species. The patterns reported herein may thus serve as taxonomic hypothesis for such taxonomic work. Further work may also help fully discern the distributional patterns for *Ligia* species and lineages in the region.

##  Supplemental Information

10.7717/peerj.4658/supp-1 Supplemental Information 1 Alignment of COI gene sequences used in phylogenetic analyses Identical sequences from different individuals were removed prior to analyses.

10.7717/peerj.4658/supp-2 Supplemental Information 2 Alignment of COI gene sequences for all sequenced individuals

10.7717/peerj.4658/supp-3 Supplemental Information 3 Alignment of NaK gene sequences for all sequenced individuals

## Additional Information and Declarations

### Field Study Permissions

The following information was supplied relating to field study approvals (i.e., approving body and any reference numbers): Field collections were carried out under Scientific Collection Permit RES2017/53 issued by the South African Department of Environmental Affairs.

### DNA Deposition

The following information was supplied regarding the deposition of DNA sequences: Sequences have been submitted to GenBank and are accessible under Accession Numbers MH173093–MH173155. Sequence data can also be found in the Supplemental Information.

### Data Availability

The following information was supplied regarding data availability: The raw datasets are provided as Supplemental File.
